# Real-life functioning and duration of illness in schizophrenia: A mediation analysis

**DOI:** 10.1016/j.heliyon.2024.e41332

**Published:** 2024-12-18

**Authors:** C. Brasso, S. Bellino, P. Bozzatello, C. Montemagni, P. Rocca

**Affiliations:** a Departement of Neuroscience “Rita Levi Montalcini”, University of Turin, Italy; b Struttura Complessa di Psichiatria Universitaria, Dipartimento di Neuroscienze e Salute Mentale, Azienda Ospedaliero-Universitaria “Città della Salute e della Scienza di Torino”, Turin, Italy

**Keywords:** Mediator, Disorganization, Metacognition, Mastery, Everyday life skills, Working skills

## Abstract

Patients with schizophrenia (SZ) often experience difficulties and impairment in daily functioning. Various factors influence functional outcomes, such as the duration of illness (DOI), the intensity of symptoms, and cognitive impairments. This study aimed at assessing the total, direct, and indirect contribution of the DOI to three key areas of daily functioning for individuals with stable SZ: life skills, work abilities, and interpersonal relationships.

Spearman's partial correlations, adjusted for age, gender, and education, were computed between the DOI, symptoms and cognitive variables, and the three real-life functioning domains. We performed three generalized linear mediation models, one for each selected domain of functioning as the dependent variable. Symptoms and cognitive variables significantly correlated with the DOI and at least one of the functioning domains were included in the mediation models as possible mediators between the DOI and the domain of real-life functioning with which they were correlated. The DOI was the independent variable in all models. Effects were computed in total, direct, indirect, and component-estimated forms. A p-value of < .05 was considered statistically significant.

A longer DOI was associated with poorer everyday life and working skills, while no such link was found with interpersonal relationships. The negative effect of the DOI on everyday life and working skills was mediated by disorganization and metacognitive mastery and disorganization alone respectively. Early targeted interventions on disorganization and metacognitive mastery might lead to improvements in the functional outcomes of people living with SZ.

## 1 Introduction

Schizophrenia (SZ) is a debilitating psychiatric condition with an estimated lifetime prevalence ranging from 0.5 % to 1 %. It is characterized by significant variability in its risk factors, clinical presentations, co-occurring disorders, responses to treatment, and long-term outcomes [[1], [2], [3]], representing the 20th cause of years lived with disability globally [4]. Despite significant advancements in both psychopharmacological treatments and psychosocial support, many patients with SZ continue to experience significant impairment in several aspects of daily functioning, which remains a major gap in their care, resulting in a substantial burden for patients, families, and the healthcare system [[5], [6], [7]]. Deficits in real-life functioning appears early in the illness and impact the individual's capacity to participate in work-related tasks, manage daily living activities, and maintain appropriate social interactions [8].

Numerous factors have been linked to the level of real-life functioning, including sociodemographic variables such as gender, age, ethnicity, and education level [[9], [10], [11], [12], [13], [14]]; the severity of psychopathology consisting of positive and negative symptoms, and disorganization [[15], [16], [17], [18], [19], [20], [21], [22], [23], [24]]; neurocognitive, social cognitive, and metacognitive impairment [[19], [20], [21], [22], [23], [24], [25], [26], [27], [28]]; individual resources like recovery style, coping strategies, resilience, self-esteem, physical health, and functional capacity [15,16,[29], [30], [31], [32], [33]]; and contextual factors related to social and work environments, including stigma and social and work opportunities [[32], [33], [34]].

Real-life functioning is also negatively affected by important variables, such as the duration of illness (DOI) [11,14,35,36], the duration of untreated illness [37], and the duration of untreated psychosis [[38], [39], [40], [41], [42], [43]].

The DOI appears to hurt several outcomes of SZ, such as the response to pharmacological treatments, symptomatic remission, reduction of suicide risk, improvement in cognitive abilities, and enhancement of functioning [14,44]. Furthermore, the DOI is related to several factors that affect the real-life functioning of individuals with SZ. In the initial stages of the disorder, a higher frequency of positive symptoms was observed, while disorganization and depression were more severe in patients with a longer DOI [45,46].

Regarding cognition, some studies have highlighted stability in neurocognitive profiles over time [[47], [48], [49]]; others have emphasized that at the onset of psychotic symptoms, cognition is within the normal range or at the lower limit of normality and becomes compromised as the disorder progresses [50,51]. Studies on metacognition suggest that this construct is more compromised in patients with a longer DOI [52,53].

On the other hand, the DOI itself is influenced by various environmental determinants that may promote an earlier onset of the condition. Examples of environmental risk factors for schizophrenia are ethnic minorities in low ethnic-density areas, childhood social withdrawal, childhood trauma, cannabis abuse, and urbanicity [54,55]. Specifically, the influence of urban settings on the onset and progression of schizophrenia has increasingly become a focus of research attention [56,57]. This association may depend on the design and features of urban spaces. In particular, the presence of green areas and well-designed public spaces may contribute to better mental health outcomes. At the same time, high-density living and poorly designed neighborhoods could exacerbate the challenges faced by individuals with schizophrenia [58].

Overall, the DOI can be considered a key determinant in influencing the course and expression of schizophrenia., with far-reaching implications for the individual's symptomatic presentation, neurocognitive profile, social cognitive abilities, metacognitive functioning, and overall real-life functioning [59]. The interplay between DOI and functional outcomes is complex: as the disorder persists over time, patients frequently face a complicated interplay of positive, negative, disorganized, and depressive symptoms, along with neurocognitive, social-cognitive, and metacognitive impairments, which can significantly affect their capacity to participate in daily tasks. Maintain meaningful relationships, and achieve their personal and professional goals.

In particular, settling if the DOI directly influences real-life functioning or if other variables, such as specific symptoms, neurocognitive, social-cognitive, and metacognitive impairments, mediate its effects may have important clinical implications. If the DOI directly impacts real-life functioning, then targeted preventive interventions aimed at reducing the DOI could be beneficial by delaying the onset of the disorder. Still, if the DOI exerts its influence through other factors, then interventions that address those specific deficits may be more effective in improving real-life outcomes for individuals with schizophrenia. Examples are rehabilitative interventions aimed at reducing those cognitive deficits that worsen with the illness progression.

Interventions targeting cognitive remediation, social skills development, and metacognitive therapy may hold promise in mitigating the detrimental effects of prolonged illness and empowering individuals with schizophrenia. Hence, it is crucial to understand the complex and evolving interplay between illness duration, symptoms, cognitive functions, and functional outcomes to develop comprehensive and patient-centered interventions that address the diverse challenges faced by those living with this complex and enduring disorder to reclaim their autonomy and improve their overall functioning and quality of life.

Based on available evidence, previous studies did not focus on the intricate connections between the DOI, symptoms severity, cognitive impairment, and real-world functioning. In this study, we utilized three generalized linear mediation models (GLMM) to examine the effect of the DOI on three critical aspects of real-world functioning relevant to individuals with schizophrenia living in the community: everyday life and working skills, and interpersonal relationships.

The employment of GLMMs expands the possibilities of mediation analysis, permitting the achievement of the objective of this research, i.e., dissecting the impact of the DOI on real-life functioning in three components: total, direct, and mediated by symptoms and cognitive impairment with the ultimate goal of finding useful information to guide targeted and personalized treatments.

## 2 Participants and methods

### 2.1 Participants

The study included consecutive outpatients with a diagnosis of SZ based on the DSM-5 criteria (American Psychiatric Association, 2013). Recruitment of participants took place over a period extending from January 2021 to June 2023 at the adult psychiatric outpatient clinic of the *Struttura Complessa Psichiatria Universitaria, Dipartimento di Neuroscienze e Salute Mentale, Azienda Ospedaliero-Universitaria “Città della Salute e della Scienza di Torino"*.

The inclusion criteria were age between 18 and 65 years and a condition of clinical stability. The diagnosis of SZ was confirmed by two experienced clinicians (C.B., C.M.) from our research group using the Structured Clinical Interview for DSM-5, Research Version (SCID-5-RV) [60]. Clinical stability was characterized by a minimum of three months without hospital admissions and/or modifications to treatment. Participants were excluded if they had a comorbid psychiatric condition meeting DSM-5 criteria or a history of severe head trauma resulting in over 48 h of coma. Comorbidities were evaluated using the SCID-5-RV.

### 2.2 Sociodemographic, symptoms, neurocognitive, social cognitive, metacognitive, and functioning assessment

Participants underwent a semi-structured interview to collect information on age, gender, educational background, and duration of illness. All individuals included in the study were receiving pharmacological treatment following the American Psychiatric Association guidelines [61].

Positive symptoms and disorganization were evaluated using the Positive and Negative Syndrome Scale (PANSS) [62], based on the model introduced by Wallwork et al., in 2012 [63]. In this study, the positive factor consisted of the items P1 (delusions), P3 (hallucinatory behavior), P5 (grandiosity), G9 (unusual thought content), and the disorganized/concrete factor of the items P2 (conceptual disorganization), N5 (difficulty in abstract thinking), and G11 (poor attention).

Assessment of negative symptoms was conducted using the Italian version of the Brief Negative Symptoms Scale (BNSS) [64]. These symptoms were grouped into an experiential factor denominated ‘avolition’, which consists of anhedonia, asociality, and apathy, and into a factor denominated ‘expressive deficit’, which consists of affective flattening and alogia [65].

Depressive symptoms were assessed using The Calgary Depression Scale for Schizophrenia (CDSS) [66]. This scale was proposed to investigate these kind of symptoms specifically among individuals with schizophrenia investigating nine items that assess various aspects of depression, including depressed mood, guilt feelings, hopelessness, self-depreciation, pathological guilt, morning sadness, early waking, suicidal thoughts, and observable signs of depression [66]. Compared to the PANSS, which may overlook certain subtleties of depressive symptoms in this kind of patients, the CDSS recognizes the unique manifestation of depressive symptoms in this clinical group [67]. This is relevant, as depression can often be overlooked or misinterpreted in individuals with schizophrenia, leading to inadequate treatment [68]. For symptom assessment by PANSS, BDNSS, and CDSS, two experienced psychiatrists (C.M. and C.B.) conducted an in-depth clinical interview with the patient lasting about 60 min.

Neurocognitive abilities were assessed with the Measurement and Treatment Research to Improve Cognition in Schizophrenia (MATRICS) Consensus Cognitive Battery (MCCB) [69,70] which uses nine distinct tests designed to measure six neurocognitive domains: processing speed, working memory, reasoning and problem-solving, attention/vigilance, visual learning, and verbal learning.

Social cognition was evaluated in terms of emotion management with the Mayer-Salovey-Caruso Emotional Intelligence Test (MSCEIT), also included in the MCCB [69,70].

The MCCB results were expressed as standardized T-scores for age and gender, with higher scores indicating better performance.

Metacognitive abilities were assessed with the Metacognition Assessment Scale (MAS) [71], which assesses four domains: self-reflection or self-awareness, the capacity to understand others' minds, decentration or the ability to understand the belonging to a larger entity, and Mastery, which involves utilizing metacognitive abilities to understand a sense life and confront its challenges.

Two experienced psychiatrists (C.M. and C.B.) underwent training to administer the PANSS, BNSS, and MAS in accordance with standardized procedures to minimize inter-rater variability. At the start of the study, they independently rated the interviews conducted with the first 20 participants. Following this, they held discussions of each interview to reach consensus on their ratings. The level of agreement between the raters (within a margin of one point) ranged from 80 % to 95 % for all PANSS items, 80 %–90 % for all BNSS items, 85 %–95 % for CDSS items, and 80 % for the MAS total score. To ensure consistency in inter-rater reliability throughout the study, the two raters participated in a quarterly review of a random sample of interviews with the final author (PR).

The Italian version of the Specific Level of Functioning Scale (SLOF) [72] was used to assess real-world functioning. This comprehensive tool consists of 43 items grouped into six subscales: physical condition, personal care abilities, interpersonal interactions, social acceptability, daily living skills, and occupational skills. The scale was completed by the patient's primary caregiver, defined as the individual in closest and most frequent contact with the patient [7,73]. For the purposes of this study, three subscales particularly relevant to individuals with SZ were analyzed: interpersonal interactions, daily living skills, and occupational skills [[72], [73], [74]].

### 2.3 Statistical analysis

An expectation-maximization algorithm was applied to impute missing data, under the assumption that this missing data occurred randomly. A total of 59 values, representing 1.4 % of the total dataset, were imputed. No variables were excluded due to a high rate of missing data. The imputation process was carried out using SPSS Statistics (IBM) version 28.0.

The Shapiro-Wilk test was used to assess whether the continuous variables followed a normal distribution. Continuous variables were reported as medians accompanied by interquartile ranges and categorical ones with absolute and relative frequencies. Spearman's partial correlations controlling for age, gender, and education were calculated between the DOI, symptoms and cognitive variables, and the three real-life functioning domains. A p-value of < .05 was considered indicative of statistical significance.

We performed three generalized linear mediation models, one for each domain of real-world functioning chosen as the dependent variable. Symptoms and cognitive variables significantly correlated with the DOI and at least one of the functioning domains were included in the mediation models as possible mediators between the DOI and the domain of real-life functioning they were correlated with. The DOI was the independent variable in all models. Total, direct, indirect, and component-estimated effects were considered statistically significant with a 95 % confidence interval (delta method) not including 0 and with a p-value <0.05. The model fits of the direct and total effect of the DOI on the three real-life functioning domains assessed were compared using adjusted R^2^ values. These values offer a reliable measure of the models’ predictive capacity while considering predictor variables, that is mediators in this case [75,76]. Descriptive statistics, correlations, and mediation models were performed with Jamovi version 2.4.11 [77,78].

Power analysis was performed with G∗Power version 3.9.1.7. For the *a priori* power analysis, we assumed a multivariate logistic regression model with 17 independent variables (symptoms, cognitive variables, and DOI), a beta error of .10, an alpha error of .05, and a medium effect size (.15). For the *post hoc* power analysis, the effect size based on the correlations between the significant predictors and between the significant predictors and the outcome variable was calculated.

## 3 Results

### 3.1 Description of the sample

We included 185 consecutive outpatients with SZ. Since continuous variables did not follow a normal distribution, they were summarized using medians and interquartile ranges (IQR). Patients were mostly males, with a median age of 36 and an intermediate-high educational level (13 median years of education). The DOI interquartile range was relatively high, namely 18 years, with a median of 8 years. Neurocognitive impairment was more evident in the speed of processing and attention/vigilance domains. The metacognitive deficit was more marked for understanding others’ minds and mastery abilities. Real-life functioning domains showed moderate impairment, which is more evident in interpersonal relationships and working skills. The characteristics of the sample are summarized in Table 1.

**Table 1:** Characteristics of the sample (n = 185).

**Sociodemographic characteristics**	
Sex, females	55 [29.7 %]
Age, years	36 (25, 45)
Education, years	13 (9, 13)
**Duration of illness**	
DOI, years	8 (2, 20)
**Severity of symptoms**	
Positive factor, PANSS composite score	9.0 (6.0, 12.0)
Disorganized/concrete factor, PANSS composite score	8.0 (6.0, 10.0)
Avolition factor, BNSS composite score	21 (12, 27)
Expressive deficit factor, BNSS composite score	14 (7, 19)
Depression, CDSS total score	2.0 (0.0, 6.0)
**Neurocognition**	
Speed of processing, MCCB T-score	23 (19, 29)
Working memory, MCCB T-score	32 (22, 39)
Attention and vigilance, MCCB T-score	29 (19, 36)
Reasoning and problem-solving, MCCB T-score	34 (28, 36)
Verbal learning, MCCB T-score	34 (27, 40)
Visual learning, MCCB T-score	35 (24, 49)
**Social cognition**	
Emotion management, MSCEIT-MCCB T-score	31 (23, 40)
**Metacognition**	
Self-reflectivity, MAS score	7 (4, 8)
Others' mind, MAS score	4 (2, 6)
Decentration, MAS score	2 (0, 2)
Mastery, MAS score	3 (1, 5)
**Real-life functioning**	
Everyday life skills, SLOF score	48 (41, 51)
Working skills, SLOF score	21 (16, 25)
Interpersonal relationships, SLOF score	23 (19, 27)

n [%]; Median (IQR); IQR: interquartile range; PANSS: Positive and Negative Syndrome Scale; BNSS: Brief Negative Symptom Scale; CDSS: Calgary Depression Scale for Schizophrenia; MCCB: Measurement and Treatment Research to Improve Cognition in Schizophrenia (MATRICS) Consensus Cognitive Battery; MSCEIT Mayer-Salovey-Caruso Emotional Intelligence Test, MAS: Metacognition Assessment Scale; SLOF: Specific Level of Functioning Scale.

### 3.2 Correlation analysis

Disorganization, avolition, and expressive deficit factors showed positive correlations with the DOI and negative correlations with the three domains of real-life functioning assessed. Speed of processing, working memory, visual learning, and all four domains of metacognition were negatively correlated with the DOI and positively correlated with the three domains of real-life functioning assessed. Correlations between all studied variables were <.7, ruling out collinearity.

Spearman's correlations between symptoms, cognition, metacognition, and real-life functioning variables are shown in Table 2.

**Table 2:** Spearman's correlations between symptoms, cognition, metacognition, and real-life functioning variables.

	DOI		Everyday life skills		Working skills		Interpersonal relationships	
**Symptoms**								
Positive factor	−0.066		−0.066		−0.078		0.007	
Disorganized/concrete factor	0.201	∗∗	−0.612	∗∗∗	−0.510	∗∗∗	−0.347	∗∗∗
Avolition factor	0.220	∗∗	−0.368	∗∗∗	−0.330	∗∗∗	−0.567	∗∗∗
Expressive deficit factor	0.245	∗∗∗	−0.387	∗∗∗	−0.315	∗∗∗	−0.505	∗∗∗
Depression	−0.015		−0.131		−0.005		−0.153	∗
**Neurocognition**								
Speed of processing	−0.165	∗	0.186	∗	0.176	∗	0.157	∗
Working memory	−0.197	∗∗	0.264	∗∗∗	0.210	∗∗	0.255	∗∗∗
Attention and vigilance	−0.088		0.217	∗∗	0.226	∗∗	0.017	
Reasoning and problem-solving	0.012		0.163	∗	0.167	∗	0.113	
Verbal learning	−0.102		0.087		0.145		0.104	
Visual learning	−0.218	∗∗	0.273	∗∗∗	0.245	∗∗∗	0.206	∗∗
**Social Cognition**								
Emotion management	−0.115		0.125		0.191	∗∗	0.234	∗∗
**Metacognition**								
Self-reflectivity	−0.243	∗∗∗	0.360	∗∗∗	0.307	∗∗∗	0.390	∗∗∗
Others' mind	−0.325	∗∗∗	0.414	∗∗∗	0.348	∗∗∗	0.392	∗∗∗
Decentration	−0.157	∗	0.268	∗∗∗	0.250	∗∗∗	0.248	∗∗∗
Mastery	−0.232	∗∗	0.481	∗∗∗	0.316	∗∗∗	0.383	∗∗∗

DOI: duration of illness; ∗p < .05, ∗∗p < .01, ∗∗∗p < .001.

### 3.3 Mediation analysis

Table 3, Table 4 summarize the mediation model with everyday life and working skills as dependent variables, respectively.

**Table 3:** Mediation model between the duration of illness and everyday life skills.

Type of effect	Effect	β	95% C.I.		p	Adj. R^2^
			Lower limit	Upper limit		
**Indirect**	*DOI ⇒ Disorganized/concrete factor ⇒ Everyday life skills*	*-.107*	*-.110*	*-.026*	*.002*	
	DOI ⇒ Avolition factor ⇒ Everyday life skills	-.005	-.016	.010	.619	
	DOI ⇒ Expressive deficit factor ⇒ Everyday life skills	-.001	-.019	.017	.944	
	DOI ⇒ Speed of processing ⇒ Everyday life skills	.016	-.011	.031	.343	
	DOI ⇒ Working memory ⇒ Everyday life skills	-.046	-.068	.010	.142	
	DOI ⇒ Visual learning ⇒ Everyday life skills	-.006	-.028	.020	.753	
	DOI ⇒ Self-reflectivity ⇒ Everyday life skills	.012	-.027	.043	.655	
	DOI ⇒ Others' mind ⇒ Everyday life skills	-.050	-.073	.010	.135	
	DOI ⇒ Decentration ⇒ Everyday life skills	-.002	-.026	.023	.908	
	*DOI ⇒ Mastery ⇒ Everyday life skills*	*-.045*	*-.057*	*.000*	*.047*	
**Component**	*DOI ⇒ Disorganized/concrete factor*	*.264*	*.034*	*.110*	*< .001*	
	*Disorganized/concrete factor ⇒ Everyday life skills*	*-.404*	*-1.260*	*-.628*	*< .001*	
	DOI ⇒ Avolition factor	.122	-.017	.204	.096	
	Avolition factor ⇒ Everyday life skills	-.042	-.166	.096	.603	
	*DOI ⇒ Expressive deficit factor*	*.169*	*.018*	*.205*	*.020*	
	Expressive deficit factor ⇒ Everyday life skills	-.006	-.166	.155	.944	
	*DOI ⇒ Speed of processing*	*-.215*	*-.250*	*-.052*	*.003*	
	Speed of processing ⇒ Everyday life skills	-.073	-.196	.064	.318	
	*DOI ⇒ Working memory*	*-.371*	*-.467*	*-.220*	*< .001*	
	Working memory ⇒ Everyday life skills	.124	-.024	.195	.127	
	*DOI ⇒ Visual learning*	*-.255*	*-.486*	*-.143*	*< .001*	
	Visual learning ⇒ Everyday life skills	.024	-.064	.088	.752	
	*DOI ⇒ Self-reflectivity*	*-.333*	*-.106*	*-.044*	*< .001*	
	Self-reflectivity ⇒ Everyday life skills	-.037	-.567	.356	.654	
	*DOI ⇒ Others' mind*	*-.331*	*-.087*	*-.036*	*< .001*	
	Others’ mind ⇒ Everyday life skills	.150	-.126	1.150	.116	
	*DOI ⇒ Decentration*	*-.275*	*-.043*	*-.014*	*< .001*	
	Decentration ⇒ Everyday life skills	.008	-.813	.915	.908	
	*DOI ⇒ Mastery*	*-.240*	*-.086*	*-.023*	*< .001*	
	*Mastery ⇒ Everyday life skills*	*.187*	*.107*	*.944*	*.014*	
**Direct**	DOI ⇒ Everyday life skills	.015	-.068	.087	.811	.043

**Total**	*DOI ⇒ Everyday life skills*	*-.219*	*-.229*	*-.050*	*.002*	.390

DOI: duration of illness; β = completely standardized effect size.

**Table 4:** Mediation model between the duration of illness and working skills.

Type of effect	Effect	β	95 % C.I.		p	
			Lower limit	Upper limit		Adj. R^2^
**Indirect**	*DOI ⇒ Disorganized/concrete factor ⇒ Working skills*	*−.114*	*−.109*	*−.025*	*.002*	
	DOI ⇒ Avolition factor ⇒ Working skills	−.007	−.018	.009	.527	
	DOI ⇒ Expressive deficit factor ⇒ Working skills	.000	−.019	.018	.983	
	DOI ⇒ Speed of processing ⇒ Working skills	.002	−.019	.021	.913	
	DOI ⇒ Working memory ⇒ Working skills	.007	−.035	.042	.844	
	DOI ⇒ Visual learning ⇒ Working skills	−.009	−.030	.019	.677	
	DOI ⇒ Self-reflectivity ⇒ Working skills	.013	−.028	.043	.685	
	DOI ⇒ Others' mind ⇒ Working skills	.007	−.036	.045	.832	
	DOI ⇒ Decentration ⇒ Working skills	−.028	−.043	.010	.229	
	DOI ⇒ Mastery ⇒ Working skills	−.026	−.040	.010	.233	
**Component**	*DOI ⇒ Disorganized/concrete factor*	*.264*	*.034*	*.110*	*< .001*	
	*Disorganized/concrete factor ⇒ Working skills*	*−.430*	*−1251*	*−.605*	*< .001*	
	DOI ⇒ Avolition factor	.122	−.017	.204	.096	
	Avolition factor ⇒ Working skills	−.061	−.181	.088	.494	
	*DOI ⇒ Expressive deficit factor*	*.169*	*.018*	*.205*	*.02*	
	Expressive deficit factor ⇒ Working skills	−.002	−.166	.162	.983	
	*DOI ⇒ Speed of processing*	*−.215*	*−.250*	*−.052*	*.003*	
	Speed of processing ⇒ Working skills	−.009	−.140	.125	.913	
	*DOI ⇒ Working memory*	*−.371*	*−.467*	*−.220*	*< .001*	
	Working memory ⇒ Working skills	−.018	−.123	.101	.844	
	*DOI ⇒ Visual learning*	*−.255*	*−.486*	*−.143*	*< .001*	
	Visual learning ⇒ Working skills	.035	−.061	.094	.675	
	*DOI ⇒ Self-reflectivity*	*−.333*	*−.106*	*−.044*	*< .001*	
	Self-reflectivity ⇒ Working skills	−.038	−.571	.374	.684	
	*DOI ⇒ Others' mind*	*−.331*	*−.087*	*−.036*	*< .001*	
	Others' mind ⇒ Working skills	−.022	−.723	.582	.832	
	*DOI ⇒ Decentration*	*−.275*	*−.043*	*−.014*	*< .001*	
	Decentration ⇒ Working skills	.101	−.313	1455	.206	
	*DOI ⇒ Mastery*	*−.240*	*−.086*	*−.023*	*< .001*	
	Mastery ⇒ Working skills	.108	−.150	.707	.202	
**Direct**	DOI ⇒ Working skills	−.072	−.122	.037	.294	.047

**Total**	*DOI ⇒ Working skills*	*−.228*	*−.217*	*−.051*	*.002*	.298

DOI: duration of illness; β = completely standardized effect size.

In both models, we found a significant negative total effect between the DOI and real-world functioning with completely standardized effect sizes (β) of −.219 between the DOI and everyday life skills and −.228 between the DOI and working skills. The model using interpersonal relationships as the dependent variable did not demonstrate a significant total effect and was not reported.

The indirect component representing the mediation of disorganization between the DOI and real-world functioning was significant in both models, while metacognitive mastery was a significant mediator between the DOI and everyday life skills only.

In both models, the DOI did not exhibit a significant direct effect on everyday life or working skills and its adjusted R^2^ was markedly lower than that of the total effect. This indicates that the DOI effect was fully mediated.

We found significant component effects between the DOI and all the psychopathological, cognitive, and metacognitive variables included as mediators except for the avolition factor of negative symptoms. In accordance with Spearman's correlations, completely standardized effect sizes (β) of the components between the DOI and symptoms were positive and those between the DOI and cognitive and metacognitive variables were negative.

**Fig. 1:**
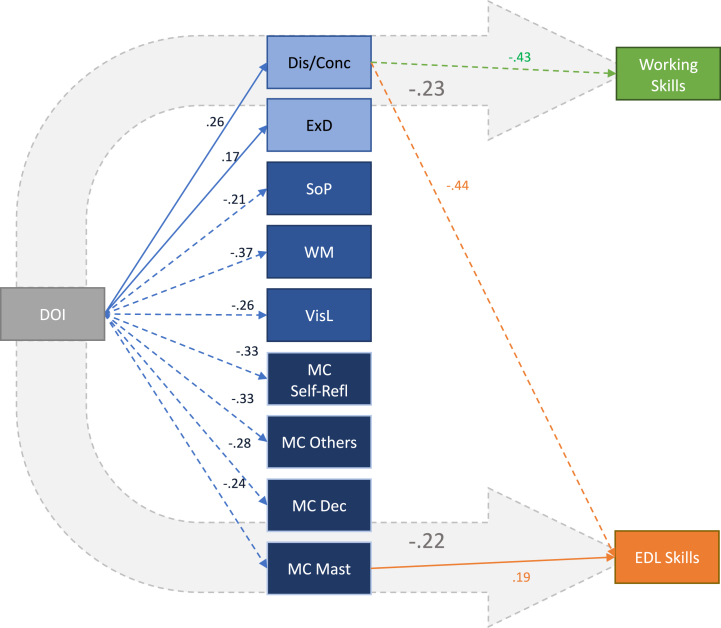
Representation of the two significant mediation models. DOI: duration of illness; Dis/Conc: disorganized/concrete factor; ExD: expressive deficit factor; SoP: speed of processing: WM: working memory; VisL: visual learning; MC: metacognition: Self-Ref: self-reflectivity; Others: Others' mind; Dec: decentration; Mast: mastery; EDL: everyday life. Symptoms are represented in light blue, neurocognitive variables in blue, and metacognitive variables in deep blue. The dotted arrows represent negative effects. The big grey arrows represent the total effect of the DOI on working skills and EDL skills. The blue arrows indicate the component effects between the DOI and the psychopathological, cognitive, and metacognitive variables, which were common to the two models. The green arrow represents the significant component effect specific to the working skills model and the orange ones those specific to the EDL skills model.

The significant components and the total effect of the two significant mediation models are shown in Fig. 1.

Regarding the power analysis, an *a priori* sample size of 179 subjects was indicated to obtain 90 % power; that of the present study is 185. The *post hoc* power analysis returned a power of 91.4 %.

## 4 Discussion

The aim of the present study was to evaluate the total, direct, and indirect (mediated) contributions of the DOI to three domains of real-life functioning relevant to outpatients with stable SZ. We found that the negative effect of the DOI on everyday life and working skills was fully mediated: disorganization mediated its impact on everyday life and working skills, while metacognitive mastery had the same role on working skills only.

First, disorganization substantially contributed to mediating the effect of the DOI on everyday life and working skills. In this study, disorganization comprised three PANSS items according to the solution proposed by Ref. [63]: ‘conceptual disorganization’, a core element of SZ, which refers to Bleuler's concept of “loosening of the associations”; ‘difficulty in abstract thinking’ that corresponds to the “concretism” also described by Bleuler; and ‘poor attention’, a cognitive symptom commonly associated with SZ [79]. These three symptoms constitute pivotal elements of the disorder from the onset to more advanced stages [[80], [81], [82], [83], [84], [85], [86]]. Two international multicenter studies on more than 2.300 patients showed a significant worsening of these three symptoms with the progression of the illness. The severity increase of disorganization slowly starts from a DOI of 3 years and becomes almost an exponential growth from about 19 years of illness [45,46]. Also, the negative relationship between disorganization and the real-life functioning of patients with SZ is well-established [6,24,87,88]. Pelizza et al. [89] found that disorganization was clinically relevant since the onset of full-blown psychosis and showed an enduring correlation with functioning deterioration. Galderisi et al. [6] demonstrated in a large cross-sectional multicenter study that disorganization had the most substantial effect on real-life functioning among ten other variables related to the illness, the context, and personal resources.

At the same time, another work from the same research network (Italian Network for Research on Psychoses) evidenced a strong and direct association between 'conceptual disorganization' and everyday life skills, 'difficulty in abstract thinking' showed a moderate association with both everyday life and work skills, while 'poor attention' was found to be exclusively linked to work skills [24]. Finally, a network analysis from our group showed a high centrality of disorganization connecting symptoms and cognition to real-life functioning both in early and late-phase SZ [87]. In the present study, the deterioration of disorganization as the illness advances explains much of the negative effect of DOI on functional outcomes. As the duration of illness increases, this symptom cluster becomes more pronounced, leading to significant challenges in maintaining employment and managing the demands of daily life [90]. Second, metacognitive mastery acts as a mediator in the influence of the DOI on everyday life skills. Mastery, a component of metacognition, defined as the ability to synthesize knowledge about one's and others' mental states, including emotion and cognition, and use it pragmatically to face life challenges [28,71]. These problem-solving strategies are multi-leveled from basic requirements to more complex, multi-tiered strategies, which necessitate a progressively articulated integration of metacognitive skills [71].

Subjects with prolonged SZ showed a significant mastery impairment as compared to patients with early psychosis or depression, indicating that the ability to apply metacognitive knowledge deteriorates as the illness progresses [52]. This progressive degradation of mastery might depend on an increasing demoralization defined as a continuous lack of coping strategies to manage daily life challenges accompanied by feelings of hopelessness, lack of purpose, a sense of incompetence, and diminished self-esteem [52,88,91]. The metacognitive mastery impairment associated with SZ was linked to poorer psychosocial functioning, specifically in everyday life problem-solving activities needing comprehension and planning [26,52,[92], [93], [94]]. We found that a longer DOI is associated with reduced metacognitive mastery that, in turn, mediates – along with disorganization – the negative relationship between the DOI and the functioning in performing everyday life activities. This result suggests a specific role of metacognitive mastery impairment in task-oriented activities of everyday life functioning, which becomes more evident with the progression of the illness. More in detail, a gradual loss of mastery skills to the point of erosion of basic requirements makes it progressively more challenging to perform daily actions such as cleaning the house, cooking, washing clothes, shopping, paying bills, using public transportation, being adherent to prescribed therapies, and utilizing properly medical and community services. Consequently, at least at its most basic levels, metacognitive mastery would constitute a fundamental element that needs to be preserved to maintain good functioning in everyday life.

Third, our study shows that the functional decline associated with the progression of the illness is significantly mediated by the worsening of disorganization and the impairment of metacognitive mastery, which in turn are related to a longer DOI. Consequently, targeted treatment to reduce disorganization and metacognitive mastery impairment might improve the trajectory of the disorder over time. Examples of effective psychosocial intervention on these illness-related variables are Metacognitive Training [95], Metacognitive reflection and insight therapy (MERIT) [96], Computerised Interactive Remediation of Cognition – a Training for Schizophrenia (CIRCuiTS) [97], and PragmaCom [98] whose application right after the onset of the disorder might have the potential to partially prevent the harmful effects of the advancement of the condition and consequently promote functional recovery.

The absence of a total, direct, and indirect effect of DOI on interpersonal functioning could result from the following factors. First, DOI might not be linked with social functioning, which instead might depend on other factors related to the patient's life context. Second, these kinds of social variables aimed at analyzing the social fabric in which the patient is embedded have not been studied and, therefore, have not been evaluated as possible mediators between the DOI and functioning. Third, the interindividual variability in interpersonal functioning might be more pronounced than the other two functional domains examined. As a result, a larger sample would be required to achieve statistically significant outcomes.

The main limitation of the current study is its cross-sectional design, which prevents the longitudinal examination of the DOI and its mediators' role in real-life functioning. Additionally, this mediation analysis does not allow us to determine the directionality of the associations between the variables, thus restricting the clinical implication of the present study. In addition, the relatively small sample size might have led to the negative result concerning the mediation model with intrapersonal relationships as the dependent variable. Another methodological weakness is the statistical models employed. Although GLMMs are generalized linear models that expand the possibilities of studying complex interactions between variables, in particular mediations and moderations, they may exhibit less robustness and stability than other models using latent variables, such as structural equation models (SEMs). Moreover, we assessed only one aspect of social cognition, specifically emotion processing, using the emotion management section of the MSCEIT. A more comprehensive evaluation of social cognition would be beneficial. Finally, since participants met the clinical stability criteria and were receiving antipsychotic treatment, our findings may not apply to acute inpatients or drug-free individuals with schizophrenia.

Although there are certain limitations, the study offers several strengths. One of the main merits is that it expanded the evidence about the role of the DOI in three main aspects of the real-life functioning of people with SZ while considering many possible mediators. Second, the assessment was performed with up-to-date instruments. Third, the study reached sufficient statistical power (>90 %) suggesting a true relationship between DOI, mediators, and everyday life and working skills.

## 5 Conclusion

The results of this study provide important insights into the complex interplay between the DOI and different aspects of functioning in people with schizophrenia. Specifically, our results suggest that the DOI in schizophrenia has a crucial impact on everyday life and work-related abilities, with longer durations linked to worse outcomes in these domains. This highlights the significance of early intervention and effective management strategies to mitigate the detrimental effects of prolonged illness on functional outcomes. The mediating role of disorganization and metacognitive mastery deficit suggests that preventive strategies should target these specific aspects of the disorder, as they appear to be crucial mechanisms underlying the link between illness duration and functional impairment.

The key lessons gleaned from this study underscore the significance of the DOI as a crucial element in comprehending the progression of schizophrenia. Specifically, the findings reveal that the adverse impact of prolonged illness duration on individuals' everyday life and working skills is entirely mediated by disorganization and metacognitive mastery. This provides valuable insights into the underlying mechanisms driving the deterioration of functional abilities throughout the illness and orients targeted rehabilitative interventions.

The main limitation of this study is represented by its cross-sectional design, which does not allow the establishment of causal relationships and the observation of longitudinal trajectories.

Future research employing longitudinal approaches would be instrumental in elucidating the dynamic interplay between the duration of illness, symptom profiles, and functional outcomes over time. Additionally, examining the potential moderating or protective elements that may reduce the negative effects of prolonged illness on real-life functioning would be a valuable avenue for further investigation.

## CRediT authorship contribution statement

**C. Brasso:** Writing – review & editing, Writing – original draft, Methodology, Data curation, Conceptualization. **S. Bellino:** Writing – review & editing. **P. Bozzatello:** Writing – review & editing. **C. Montemagni:** Writing – review & editing. **P. Rocca:** Writing – review & editing, Methodology, Conceptualization.

## Ethics approval and consent to participate

This study was reviewed and approved by the Comitato Etico Territoriale Interaziendale AOU Città della Salute e della Scienza di Torino with the approval number: 0126325, dated December 20, 2019.

All patients or their legal guardians provided written informed consent to participate in the study and for their data to be published.

## Availability of data and materials

Due to the anonymity ensured in the informed consent documents at the time of data collection, the data cannot be shared publicly and are under the control of the Comitato Etico Interaziendale of the A.O.U. Città della Salute e della Scienza di Torino. Researchers interested in accessing these data may reach out to the corresponding author (claudio.brasso@unito.it).

## Declaration of competing interest

The authors declare that they have no known competing financial interests or personal relationships that could have appeared to influence the work reported in this paper.
